# Evidence on the Health Benefits of Supplemental Propolis

**DOI:** 10.3390/nu11112705

**Published:** 2019-11-08

**Authors:** Andrea Braakhuis

**Affiliations:** Faculty of Medical Health Science, The University of Auckland, Auckland 1010, New Zealand; a.braakhuis@auckland.ac.nz

**Keywords:** bioactives, nutrition, cardiovascular, stroke

## Abstract

Propolis is a honey-related product with reported health benefits such as improved immunity, lowered blood pressure, treated allergies and skin conditions. A literature review and narrative synthesis were conducted to investigate the evidence on the reported health benefits and future direction of propolis products. Using a predefined search strategy we searched Medline (OvidSP), Embase and Central for quantitative and qualitative studies (1990–2018). Citation, reference, hand searches and expert consultation were also undertaken. Studies of randomised control trials and observational data on humans with health-related outcomes were included. Collected data were entered into NVivo software (Version 12, QRS International) and analysed using a thematic framework and a narrative synthesis of emergent themes. A total of 63 publications were discussed. The majority were cell-based and animal studies, with a few key human trials conducted. There is significant promise for propolis as an effective antioxidant and anti-inflammatory agent with particular promise in cardiometabolic health.

## 1. Introduction

Propolis, referred to as bee glue, is produced by bees in the construction and maintenance of their hives. Bees produce propolis using a combination of beeswax and saliva, where it acts as the defence mechanism for the hive [1]. To date, propolis has been extensively researched in thousands of scientific papers on its bioactivity and health benefits [2,3,4,5]. The protective immune defence and antioxidant properties of propolis come from the bioactive phytochemicals’ constituents. Multiple compounds have been identified in propolis and differ based on the location of production. The compounds in propolis include phenolic acids, flavonoids, esters, diterpenes, sesquiterpenes, lignans, aromatic aldehydes, alcohols, amino acids, fatty acids, vitamins and minerals [6]. The exact composition of a propolis sample will vary between hives, location and seasons. Given that the function of propolis is to support the sterility and health of the beehive, the protective properties of the bioactives found in propolis can provide significant benefits for human health [2,3]. Propolis has attracted attention in recent years due to its reported benefits, which make it a potential preventive and therapeutic agent. The sales of propolis products is growing, and in many commercial settings supply cannot keep up with demand [7]. Sales on propolis containing oral health and wound care products appear at the top of the category list [7].

The proposed health benefits most thoroughly investigated include anti-microbial, wound healing, cardio-protective and support of optimal neural function. The mechanism by which propolis supports health appears related to its antioxidant and inflammatory activity, although in most cases the range and scope of physiological effects of this complex nutrient are wide and varied.

The aim of the current review is to investigate the health properties of propolis and direct the future scope of research and discuss possible clinical implications. A mixed methods approach to this review was undertaken to avoid reporting bias and present a balanced summary of the current literature.

## 2. Methods

The authors adopted a mixed approach to the review, in particular the data collection and synthesis, to allow a range of themes to be explored, rather than pre-empting outcomes.

### 2.1. Search Strategy and Selection Criteria

Multiple search strategies were utilised to search all the current literature on health benefits of propolis. Three electronic databases were searched (Embase, Medline (OvidSP), Central) using a predefined search strategy, optimised for each database by using Medical Subject Headings and Boolean operators. The search terms were submitted to each database as described in the search strategy. The search was saved within the database with each database screened separately for inclusion by title/abstract. (Supplementary Data S1, Table S1). The included papers were exported into Endnote, duplicates removed, full text articles collected, and full text search conducted. The search was conducted in March 2018. The inclusion criteria were: propolis research published between January 1990 and March 2018; any research design; original empirical data; any aspect of health related to propolis or its polyphenol constituents; human and cell studies were included. Only English language articles were included. All research design types were considered, including randomized control trials, case studies and cell study investigations. All data sources were required to be peer reviewed published journal articles. Review articles were excluded on the basis that they may bias the current review process.

The searches produced an initial 3936 titles, with 3835 remaining after duplicates were removed. The abstracts of 3835 publications were screened by one researcher and after 177 papers remained. The full article and their included reference list were sourced for the remaining 177 publications. These 177 papers were then reviewed independently by two researchers against the inclusion criteria and the final number of included studies was 63. See Preferred Reporting Items for Systematic Reviews and Meta-analyses (PRISMA) chart (Supplementary Data S1, Figure S1).

### 2.2. Data Analysis

A thematic synthesis as described by Thomas and Harden [8] was conducted using NVivo (QRS International). The final 63 articles were exported from Endnote into NVivo. One investigator performed line-by-line coding of the findings for all included studies, conceptualising the data and inductively identified concepts. The final coding was reviewed and the node network was checked by a research assistant; discussion between investigators was conducted for any misalignment. As part of the reviewing, a word query of total data was conducted and compared to node synthesis. Visual inspection of the word query aligned well with node terminology. Similar concepts were grouped into themes and subthemes following a cluster analysis to identify similarities.

## 3. Results

Following the qualitative analysis of the 63 articles, the key themes were identified. 

The studies included in the current review range from method papers describing extraction techniques, cell-based and human interventions. The vast majority of papers were cell-based assays, using increasing dosage of propolis constituents. The human interventions discussed were generally designed as controlled trial, parallel investigations of a small sample size (*n* = 10–70) with a mixture of sick and healthy individuals.

Of the included 63 studies, 12 originated from Europe, 7 from South Asia, 30 from China, 4 from Brazil, 6 from Japan, 2 from Korea, 1 from Africa and 1 from Malaysia.

There were 6 human trials (118 healthy humans, 32 diabetic humans), 27 animal studies (790 rats, 420 mice), 24 purely cell-based assays, 2 cell and animal studies combined (24 mice) and 4 propolis compound identification studies to underpin the discussed literature. The predominant outcomes for each node or topic area are included in Table 1.

For the purposes of this review the following topics were selected for discussion, given the majority of data is presented here: physiology, digestion and metabolism; neuroprotection; atherosclerosis; immunomodulatory; wound healing and skin protection; identified subthemes included anti-microbial; free radicals and oxidative stress; cardio-protective activity; dose and safety and propolis characterisation. Each topic will be discussed below.

## 4. Discussion

### 4.1. Structure and Function of Propolis

#### 4.1.1. Characterising Propolis

Propolis is a plant-derived substance collected by honeybees from various sources and contains multiple polyphenolic constituents, mainly flavonoids and phenolic acids [64]. Propolis originates from a variety of countries including Argentina, Brazil, China, New Zealand, South Africa, Taiwan, Ukraine, United States and Uzbekistan with different geographical characteristics, and is categorised into three major classes depending on its predominant colour, either green, red or brown [28,65,66]. The different classifications and source of origins make it difficult to extrapolate health-related claims on any one propolis source [67].

Propolis is derived from animals but is contains bioactives derived from plant-based diet of the bees. Multiple compounds have been identified in propolis from different locations, including phenolic acids, flavonoids, esters, diterpenes, sesquiterpenes, lignans, aromatic aldehydes, alcohols, amino acids, fatty acids, vitamins and minerals [6]. The botanical source of origin is highly varied, which is consistent with the native flora by which the bees have access. The number of constituents identified in any individual propolis samples is in the hundreds, depending on geographic origin [10,28,68]. Whether the propolis extract was prepared with ethanol or water also changes the constituent nutrient profile [10]. The variety of compounds and phytonutrients found in propolis somewhat explains the range of biological properties reported, from antimicrobial, antioxidant, anti-inflammatory, immune modulating, skin healing, skin aesthetic, cancer protection and anti-cavity among others [69].

Of note, propolis contains a wide variety of natural phenolic compounds, mainly flavonoids and phenolic acids which have been attributed to its successful use as an anti-inflammatory and healing agent [28,70]. The pharmacological activity of flavonoids is mainly due to their structural characteristics as tricyclic compound, and to the presence of radicals attached to their rings [6]. The polyphenolic content of various propolis samples varies significantly, ranging from 143 to 324 mg gallic acid equivalents/g and from 206 to 705 mg quercetin equivalents/g of ethanolic extracts of propolis, respectively [66]. With the great diversity of propolis sub-groups, classified according to their physico-chemical composition, however there is also a large variance in the content of flavonoids [25].

According to the HPLC analysis, the phenolic content of propolis is mainly composed of crysin, galangin, pinostrobin, pinobanksin and pinocembrin, the last being the most abundant flavonoid in propolis [12,66]. European and Asian propolis contain simple phenolic acids while lignans are the main compounds in propolis from warmer climates (Brazil) [1]. A study of New Zealand propolis found 70% of the flavonoid content was pinobanksin and pinocembrin [71], much higher than samples from Brazil, Uruguay and China [72]. The differences in structure and function of the various propolis products does cloud the ability to recommend clinical and research direction. In general health claims are likely to be isolated to a product generated from a particular geographical location.

#### 4.1.2. Physiology, Digestion and Metabolism of Propolis

Propolis comprises of lipids, waxes and resins in a complex matrix with a large molecular weight, contributing to a poor bioavailability and absorption. The form of polyphenol administered (natural fruit, juice or extract) or the presence of multiple polyphenolics support synergistic effects and are important determinants of bioavailability [73]. Factors believed to contribute to poor bioavailability of polyphenols include digestive instability, poor transcellular efflux in intestinal cells, and rapid metabolism and excretion [13]. Since dietary polyphenols exist as esters, polymers or in glycosylated forms, they cannot be absorbed and must be hydrolysed by the intestinal enzymes or by the colonic microflora before absorption [73]. When in the intestinal system, poorly absorbed polyphenolic compounds are converted to smaller phenolic acids with improved bioavailability, aided by enzyme activity of the colonic microbiota [39]. As microbiota vary between people the inter-individuality in absorption and metabolism is being increasingly considered. Despite poor absorption percentages of bio-accessible phenolic compounds in propolis, the recovered amounts detected in plasma were still high due to their high initial contents compared to other food materials such as fruits and vegetables [66].

Once the propolis phenols reach the blood stream, their selective permeability across the blood–brain barrier and systemic elimination limit the therapeutic efficacy with regards to optimal brain function [73]. However, increasing evidence suggests certain propolis derivatives are capable of crossing the blood–brain barrier. Caffeic acid phenethyl ester (CAPE) is hydrolysed to caffeic acid within six hours of reaching the plasma [23,40] and CAPE has recently been shown to cross the blood brain barrier at least in rats [23,40]. The degree to which propolis polyphenols and metabolised derivatives are capable of crossing the blood–brain barrier depend on their lipophilicity with less polar polyphenols or metabolites (i.e., O-methylated derivatives) capable of greater brain uptake than the more polar ones (i.e., sulphated and glucuronidated derivatives) [74].

The rate at which polyphenols are excreted in the urine appears to vary considerably between individuals. Those placed on a three-day high phenolic diet demonstrated this variable excretion with one participant excreting 8 of the 17 phenolic acids measured, another all 17 [39]. The reasons for individual variability in excretion are generally unknown, but may be related to ageing, renal function or inherent propolis properties. Ongoing research and overall consideration to the general health of consumers of propolis and the effect on renal function is warranted.

#### 4.1.3. Dose and Safety

Clinical investigation in mice and humans report that propolis and its constituents are generally well tolerated and non-toxic unless administered in very large quantities [1,28,30]. However, it should be noted that the adverse events and toxicity of propolis are rarely included as an outcome measure in human trials. Considered a “mere” herbal product, consumers rarely consider the potential adverse side effects of nutritional supplements that are perceived to be naturally derived.

A case report of a 59-year-old man with cancer of the bile duct was supplementing with propolis. The patient developed acute renal failure and was undergoing concurrent haemodialysis therapy. Fortunately, his renal function improved following the removal of propolis. However, based on the uncertainty of propolis as the cause of renal failure at that time, the patient continued ingesting propolis for the underlying cancer. Renal function deteriorated again after re-challenge and recovered following secondary withdrawal of propolis [75]. While renal biopsies where not taken in this particular case, concerns over the adverse effects of propolis on a vulnerable population are potentially warranted. It has been suggested that CAPE, a constituent of propolis, inhibits inducible nitric oxide synthase (iNOS) pathways which may decrease kidney perfusion and thus induce acute renal failure in at-risk patients [75].

Based on previous animal studies and applying a safety margin when extrapolating to generally healthy humans, a safe dose of propolis has been reported to be 70 mg/day [27]. Interestingly, studies on pinocembrin, a component of propolis, have been conducted using 150 mg as a single dose [30]. When provided to conscious mice, the median lethal dose of propolis extract is more than 7.34 g/kg (LD50), confirming the product is generally safe. [42,43]. The difficulty with prescribing an accurate dose of propolis based on the investigated population, dosing regimens, compliance and product purity, is pronounced. As the phenolic compounds present within propolis vary based on geographical origin, the bioactivities will also vary significantly making it difficult to define a correct dosage [69].

A more common adverse effect of propolis administration is hypersensitivity, particularly in regard to topical application, resulting in allergic reactions, swelling, dermatitis and urticaria. In specific cases, an individual had severe swelling of the throat and anaphylactic shock upon topical application [44]. Severe adverse effects, such as laryngeal edema and anaphylactic shock, seldom have been reported [75], which are rarely attributed to a “natural” supplement like propolis. It is reported that 1.2–6.6% of individuals with dermatitis are sensitive to propolis [76]. As such, despite the positive safety profile, it is recommended people seek medical advice before supplementing or applying propolis products.

### 4.2. Oxidative and Inflammatory Effects of Propolis

#### 4.2.1. Free Radicals and Oxidative Stress

The antioxidant activity of propolis and its constituents has been well documented [47], with the vast majority of outcomes demonstrating a reduction in oxidative stress markers [36]. In order to reduce the oxidative stress-induced tissue damage, endogenous antioxidant systems have developed protective mechanisms including enzymes, superoxide dismutase (SOD), glutathione peroxidase (GPx) and catalase (CAT), and antioxidant nutrients ascorbic acid, glutathione and flavonoids [28]. Malondialdehyde concentrations are commonly being used as potential oxidative stress biomarkers and indicators of oxidative lipid damage. The chemical structure of the constituent polyphenols enable propolis to effectively eliminate free radicals [31]. The flavonoids in propolis are powerful antioxidants, capable of scavenging free radicals and thereby protecting the cell membrane against lipid peroxidation [13].

The most important organ of the central nervous system, the brain, is more sensitive to free radical-induced damage because of its high use of oxygen, its high concentration of polyunsaturated fatty acids, and its low concentration of antioxidant molecules compared to other tissues [27]. In the brain, oxidative stress results in acute and chronic injury and plays an important role in the pathogenesis of neuronal damage. Propolis and its derivatives appear to prevent oxidative stress in radiation-injured brain tissue by decreasing the formation of lipid peroxidation and increasing the antioxidant enzyme activities, and also by inhibiting free radical generation [27]. These results suggest an important role of propolis and its constituents as an antioxidant and free radical scavenger on the oxidative stress in the radiation-injured brain tissue. Preclinical studies have also suggested that pinocembrin, a component of propolis, protects rat brain against oxidation and apoptosis induced by ischemia-reperfusion both in vivo and in vitro [30].

In transgenic mice at risk of Alzheimer’s, those given pinocembrin maintained adequate glutathione content and SOD activity [18]. Administration of brown propolis from Iran ameliorated neuronal damage of permanent cerebral artery occlusion in mice because it restored the antioxidant SOD and GPx activity, and enzyme ratio, as well as reduced lipid peroxidation [28].

Oxidative stress is a well-known cause of persistent chronic inflammation due to its ability to activate transcription factors such as nuclear factor kappa-light-chain-enhancer of activated B cells (NF-κB), activator protein 1, tumour protein, hypoxia-inducible factor 1-alpha, peroxisome proliferator-activated receptor gamma and nuclear factor erythroid 2-related factor 2 (Nrf2) [3]. NF-κB is a protein complex that controls the transcription of DNA and is involved in cellular responses to stimuli such as stress, cytokines, free radicals, ultraviolet irradiation, oxidised low density lipoproteins (LDL) and bacterial or viral antigens. Propolis is able to activate the Nrf2 transcription factor which is a major regulator of antioxidant proteins. The binding of Nrf2 to the antioxidant response element leads to transcription of several antioxidant enzymes, including heme oxygenase-1, the regulatory and catalytic subunits of γ-glutamate-cysteine ligase, GPx, glutathione reductase, CAT, SOD and glutathione-S-transferase [33]. The clinical implications for the antioxidant activity of propolis are difficult to ascertain, however below we discuss the anti-inflammatory and neuroprotective properties of propolis, both which have an antioxidant basis.

#### 4.2.2. Anti-Inflammatory

Inflammation is defined as an interaction between the immune system and injured tissues designed to restore homeostasis via complex signalling pathways [77]. The anti-inflammatory activity of propolis appear related to its associated constituents: flavonoids, phenolic acids and their esters, terpenoids, steroids and amino acids, with CAPE being the most studied compound. The main mechanisms underlying the anti-inflammatory activity of propolis include: (1) the inhibition of cyclooxygenase (COX) and consequent inhibition of prostaglandin biosynthesis, (2) free radical scavenging as discussed below; (3) inhibition of nitric oxide synthesis; (4) reduction in the concentration of inflammatory cytokines; and (5) immunosuppressive activity [2].

COX is an enzyme that is responsible for the formation of prostaglandins, prostacyclin and thromboxane. Pharmacological inhibition of COX can provide relief from the symptoms of inflammation and pain [2]. CAPE inhibits the activity and expression of COX-2 in human oral epithelial cells and in rat models of inflammation [78]. In transgenic mice at risk of Alzheimer’s, three months of pinocembrin treatment confirms significant reductions in neuronal inflammatory markers (tumour necrosis factor alpha (TNF-α), interleukin-1 (IL-1) and interleukin-6 (IL-6) [17].

Pinocembrin reduces the level of proinflammatory cytokines (TNF-α, interleukin-1beta (IL-1β)), chemokines (intercellular adhesion molecule-1, vascular cell adhesion molecule-1), inducible nitric oxide synthase (iNOS), and aquaporin-4 [4]. In a study on rats with non-alcoholic fatty liver disease, 200 mg/day propolis reduced TNF-α and IL-6, with effects attributed to the anti-inflammatory activity of propolis [16]. Pinocembrin appears to suppress the nuclear translocation of NF-κB and decrease TNF-α expression in the cerebral cortex and the hippocampus of diabetic mice, suggesting propolis alleviates cognition deficits by protecting neurons from inflammation injury [11].

Pinocembrin may reduce damage induced radiation by the reduction of oxidative stress, inflammatory and apoptotic markers [5]. Of significance, pinocembrin normalised the infarct size elevated by radiation. The anti-inflammatory actions of pinocembrin has been evidenced by the reduced expression of IL-1b, TNF-a and iNOS [5].

Not all studies report a reduction in inflammatory markers. In type-2 diabetic patients, TNF-α was significantly decreased after the treatment with Brazilian green propolis, however IL-1β and IL-6 were significantly increased [22]. Propolis, as well as some of its components, actively stimulated the secretion of several cytokines, including IL-1β and the pro-inflammatory effects resulted from increasing IL-1β production were likely counteracted by the anti-inflammatory effects of IL-6 [22]. Therefore, the combined action of Brazilian green propolis on chronic inflammation was favourable in those with type-2 diabetes.

#### 4.2.3. Atherosclerosis and Cardio-Protective Activity

The cardiovascular effects of propolis have been widely reported, although the underlying mechanisms have been poorly characterised. Results from research conducted tend to indicate that cardio-protective effects are the result of the antioxidant activity of propolis and its constituent compounds [79]. Epidemiological studies revealed that a diet rich in flavonoids is positively correlated with increased longevity and decreased incidence of cardiovascular disease [70]. Oxidative stress appears to play a role in the initiation of cardiovascular disease. Low density lipoprotein oxidation triggers the process of atherogenesis, which is the developmental process of plaques on artery walls, resulting in atherosclerosis (a thickening of artery walls and restriction of blood flow), and finally cardiovascular disease [40]. Beyond the well-recognised anti-oxidant effects, polyphenols interact with the generation of nitric oxide (NO) from vascular endothelium, which results not only in vasodilatation, but also to the expression of genes which protect the cardiovascular system [79].

Mice on a cholesterol-rich diet revealed that all propolis types (red, green or brown) diminished total cholesterol and elevated high density lipoprotein-cholesterol concentrations, and sclerotic legions in mice, suggesting trivial bioactive differences in regional product sourcing [15]. Polyphenols are known to inhibit numerous cellular enzymes like lipoxygenase, xanthine oxidase and phospholipase A2 and reduce LDL-peroxidation. In addition, flavonoids found in propolis are iron chelators and can therefore decrease the iron-dependent production of free radicals [80]. The good scavenging activity of propolis flavonoids requires the presence of a catechol moiety in the B-ring along with 3-OH moiety in combination with a 2–3 carbon double bond in the C-ring of flavonoids, resulting in increased iron chelation and inhibiting the rate of lipid peroxidation [80]. Thus, chelation raises the level of scavenging activity of flavonoids [80]. Pinocembrin, in particular, is thought to influence cardiovascular diseases based on its ability to regulate ApoE and reduce rho kinase [4]. Propolis also showed to be protective against cerebral occlusion in mice with decreased infarct volume and improved behavioural function [28].

With regards to a particular cardiovascular-related disease, stroke, dietary polyphenols may provide benefits for different phases of the disease. It has been suggested that a nutrient timing approach could be applied to the use of propolis and other polyphenols in the reduction of cardiovascular disease. During stages of disease prevention, polyphenols improve cerebral blood flow, prevent platelet aggregation and thrombosis and inhibit oxidative stress. For the early disease stage, polyphenols reduce inflammation and protect endothelial phase, thus interfering with ischemia death mechanisms such as apoptosis and necrosis [79]. The utilisation of polyphenols like propolis in targeted disease states may be worthy of follow up in future research to provide clinical insight around optimal use.

#### 4.2.4. Neuroprotection

Traumatic neural dysfunction such as ischemia and epilepsy, or degenerative dysfunction, such as Parkinson disease, Alzheimer disease and multiple sclerosis, have had propolis interventions. The mechanisms and causes of neurological dysfunction remain elusive, however they appear linked to an increase in oxidative stress, induction of inflammatory signalling and slow immune responses in the brain tissue [45,69]. While the general antioxidant activity of propolis has been discussed, here we focus on the protective properties towards neurons and related physiology. By far the highest proportion of research on the health benefits of propolis has focused on this topic.

Recent studies have shown that CAPE, discussed earlier, acts as an antioxidant assisting in the protection of brain injury following cerebral ischemia [81]. Neurons subjected to glutamate-induced toxicity were protected by the addition of CAPE by inhibiting phosphorylation of p38 and caspase-3 activation, further supporting its neuroprotective activity [68]. Caspase-3 is a frequently activated death protease, catalysing the specific cleavage of many key cellular proteins including neural and retinal cells. CAPE also prevents neurotoxic effects due to excessive inflammatory reaction in the brain [68]. In addition, one of the propolis flavonoids, pinocembrin, reduces injury from cerebral ischemia, most likely via antioxidant activity of the nutrient [26,56].

Pinocembrin treatment of neuronal cells inhibited the increase of gene and protein levels of the pro-apoptotic gene (bax) following glutamate exposure, without affecting gene and protein expression of the anti-apoptotic gene bcl-2 [26]. Bcl-2 gene and protein expression were not altered before and after glutamate insults, which was consistent with previous research [46]. Pinocembrin provides neuroprotection, in part, by inhibiting the release of cytochrome c from the mitochondria into the cytoplasm and by downregulating the synthesis of pro-apoptotic Bax, this action may be due to the inhibition of p53 expression [56].

Alongside the research on neuronal disease, increasingly a focus on the protective effects of propolis on retinal cells is being reported [35,49]. Propolis has neuroprotective effects both against in vitro retinal damage in cell cultures, studied using induced oxidative damage and neurotoxicity in retinal cell culture, and against retinal damage in mice [10]. The implications for propolis as a preventative strategy for various eye conditions such as macular degeneration in an ageing population and myopia in the younger generation is worthy of investigation. Myopia, or short sightedness currently affects 10–20% of secondary school students and numbers affected are on the rise [82]. Evidently screen time, outdoors exposure and physical activity influence the risk of developing myopia, as might antioxidant supplements high in flavonoids, such as propolis. While myopia is currently thought of as a condition affecting Asian children, rates in the US are rapidly increasing [82]. Within many Asian families, non-drug related treatment options of myopia are sought after and represent a commercial opportunity for a product such as propolis.

### 4.3. Immune, Gut and Healing

#### 4.3.1. Immunomodulatory and Antimicrobial Function

All propolis types, irrespective of origin and consequently the compounds they contain, have shown microbial activity indicating it is rather the collective of propolis compounds rather than the individual compounds that result in its antimicrobial activity [36]. Compounds within propolis appear to prevent bacterial cell division and cause dysfunction within the cytoplasm, thereby inactivating bacterial activity and growth. Propolis has demonstrated various antibacterial activity against a range of pathogens including *Bifidobacterium infantis*, *Enterococcus faecalis*, *Escherichia coli*, *Helicobacter pylori*, *Listeria monocytogene*, *Neisseria gonorrhoeae*, *P. larvae*, *Staphylococcus aureaus*, *Staphylococcus epidermides*, *Streptococcus pyogenes* and Vancomycin-resistant *Enterococcus faecium* [1,4,24,25,70,83].

The flavonoid constituents of propolis are associated with antibacterial activity, the prominent ones including galagin, pinocembrin and pinostrobin [1]. These flavonoids are reported to increase the bacterial membrane permeability and inhibit bacterial genetic coding [1]. Further, flavonoids have been reported to inhibit nucleic acid synthesis, attachment and formation of biofilms and energy metabolism of bacteria [23] and as such demonstrate effective anti-bacterial agents.

Similarly to antibacterial activity, there is a range of reported mechanisms for propolis’ antiviral activity, however research has been limited to investigating CAPE and other related compounds [1]. CAPE blocks the NF-kB activation process [32], and is an inhibitor of HIV-1 integrase produced by retroviruses, therefore it inhibits the integration of genetic material into the host’s DNA cell. In addition, CAPE also supresses in vitro replication of hepatitis C virus [1], thus demonstrating both antibacterial and viral effects.

The commercial implication of the antibacterial action of propolis has led to the development of oral sprays, toothpaste and lozenges currently available. The efficacy of oral products over standard medical options is debateable, regardless of the antibacterial promise of propolis.

#### 4.3.2. Gut Health

The investigation of optimal gut health and gastrointestinal bacterial characterisation is quite topical, with some suggesting disease states are caused by imbalances in the gut microbiota [84]. A healthy array of intestinal microbiota releases microbial bioactive molecules, such as short chain fatty acids, which confer particular health benefits. Polyphenols, including propolis constituents, have recently been defined as probiotics by an international consortium as they are selectively metabolised by gut microbiota [55]. Propolis polyphenols may support optimal gut microbiota by inhibiting the growth of pathogenic bacteria and suppressing the adhesion of gut pathogens to human gut cells [39].

In a recent study Alkhaldy et al. reported propolis derived phenolics appear to protect the gastrointestinal tract by inhibiting the growth of pathogenic bacteria such as *Clostridium* spp, *Staphylococcus aureus* and *bacteriosides* spp. [39]. In addition, Alkhaldy et al. reported propolis suppressed the adhesion of pathogens to the intestinal wall and enhanced systemic immune function including natural killer cell activity and cytokine secretion [39].

Despite the recent reported effects of propolis and its probiotic nature, theories have been proposed that it may have an equal and opposite inhibition of microbial growth. Haddadin et al. demonstrated lower bifidiobacteria spp. numbers in vivo when grown with propolis, however the bacteria present were capable of producing greater amounts of short chain fatty acids particularly butyric acid [24]. Perhaps the activity of the existing bacteria is increased in the presence of propolis. Overall the degree that propolis supports the health of the intestinal microflora has been poorly investigated with longer term studies needed on the effect of dietary polyphenols on gut microbiota, an area worthy of further research.

While the use of propolis as a source of nutrition for gut bacteria has been discussed, the effect on intestinal barrier function has also been investigated. A gut cell line treated with propolis showed activated adenosine monophosphate-activated protein kinase (AMPK), extracellular signal-regulated kinase ½ (ERK), p38, and protein kinase B [64]. Rats fed a diet supplemented with propolis exhibited increased colonic epithelium ZO-1 expression [64], suggesting it strengthens intestinal barrier function by activating AMPK and ERK signalling. Studies on the effect of propolis on gut microbiota provide novel insights into the potential application for human health, particularly conditions such as inflammatory and ulcerative colitis. While there is potential for propolis to support optimal gut health, the research is in its infancy and requires more research before recommending clinically.

#### 4.3.3. Wound Healing and Skin Protection

The antioxidant effects of propolis have been discussed previously, acknowledging the effect oxidative stress has on skin and wound healing as an important clinical application. Historically propolis has been used as a skin disinfection and directly on wounds to improve healing [24,70] with modern medicine taking up the investigative cause. Propolis has been reported to stimulate the growth of skin tissue and regeneration as well as modulate collagen production [1,31]. Burn wounds treated with propolis were found to have lower concentrations of free radicals [85]. Propolis has been reported to reduce oxidative stress in wounds by inducing the expression of antioxidant related genes (hemeoxygenase-1 (HO-1), glutamate-cysteine ligase- modifier GCLM and- catalytic GCLC subunits), and improve collagen expression and cell viability on cells exposed to significant oxidative stress [31]. In vitro investigation demonstrated the addition of propolis could alleviate cell damage in fibroblast cells by suppressing intracellular free radical production induced by excessive light [31]. While direct antioxidant benefits have been discussed, the anti-inflammatory effects of propolis have also been shown to improve wound healing [40].

The geographical origins, colour and composition of the propolis can affect this protective mechanism with green propolis being more effective at wound healing in rats than red propolis [52]. Weleda (New Zealand) Ltd. successfully gained New Zealand Medicines and Medical Devices Safety Authority to use propolis as an ingredient in topical products aimed at patients with eczema [86], suggesting the product is safe to use on skin. The range of propolis-containing skin products available commercially is expanding with creams and lotions predominating. The advertising avenue taken by a majority of skin products claims a “calming, moisture rich, anti-ageing” effects. Very few point to the potential wound healing properties that appear to have some research efficacy.

## 5. Conclusions

As a natural compound with good pharmacological and pharmaceutical properties, propolis and its constituents have a wide application, including wound and skin healing, neurodegenerative disease and atherosclerosis. There is certainly an interest in the health properties of propolis and a subsequent growth in publication history since 1990, however a need for more clinical trials are sorely needed to confirm the value of propolis to a specific population. While a greater number of human studies are warranted, certainly the preclinical data supports a role in antioxidant and anti-inflammatory activity of propolis which supports a reduction in various chronic diseases including heart disease, diabetes, hypertension and neuronal degenerative disease such as Alzheimer’s. An interesting avenue and possible future commercial direction could investigate the effect of propolis on cardiovascular health.

## Figures and Tables

**Table 1:** Summary of included studies derived for each theme.

References	Theme Citation Count	Emergent Themes and Key Outcome Measures
[9,10,11,12,13]	34	Anti-Cancer and Anti-Tumor Effects. Outcomes: Cancer cell culture response to propolis (apoptosis, regulatory markers, cancer cell formation, angiogenesis); apoptosis in brain cancer cell lines by propolin G (propolis component); apoptosis and caspase-3 in ischemic rats given pinocembrin; caspase 3 and cytochrome C in ischemic rats
[11,12,14,15,16,17,18,19,20,21,22]	53	Anti-Inflammatory Activity. Outcomes: cell line inflammatory response to propolis constituents; oxidative stress in rat cortical neurons; IL-6 and TNF-α in rats with fatty liver disease given propolis; inflammation in injured human brain cells; TNF-α and NFkb in diabetic mice; inflammation in diabetic humans
[20,23,24,25]	33	Antimicrobial and Antiviral Effect. Outcomes: Bacterial growth in propolis agar; parasite infected rats administered propolis; bacterial plaque and pathogenic microflora in the human mouth
[13,15,26]	35	Cardioprotective Activity and Atherosclerosis. Outcomes: Atherosclerotic legions in propolis treated mice; total cholesterol, HDL, ALT and ALP in rats with fatty liver disease given propolis; infarct volume in ischemic rats given pinocembrin; mitochondrial function in mice given pinocembrin
[12,14,15,19,21,22,26,27,28,29,30,31,32,33,34,35,36,37,38]	51	Characterising Propolis: Outcomes: Propolis compound identification; constituents of propolis-pinocembrin, propolin G; geographical location and source; flavonoid interactions
[13,29,30,39,40,41]	22	Digestion and Metabolism. Outcomes: Bioavailability; taste, sensory and bioactivity of propolis in humans
[14,15,20,27,28,30,31,32,42,43,44]	27	Dose and Safety. Outcomes: Tolerability of pinocembrin in humans; propolis induced no toxicity as determined by the ALT, AST and CK plasma levels in parasitic mice; edema, side effects, topical application; generally non-toxic in mice
[4,9,10,12,13,15,16,17,19,21,22,23,27,28,30,31,32,33,35,37,38,45,46,47,48,49,50,51,52,53,54]	151	Free Radicals and Oxidative Stress. Outcomes: MDA, lipid peroxidation and antioxidant enzymes in homogenized brain tissue of irradiated rats and cerebral-injured mice; superoxide production, glutathione depletion and intracellular superoxide burst following propolis intake in humans; mitochondrial response of neuroblastoma cells to propolis treatment; neurotoxicity, apoptosis and oxidative stress in cultured retinal ganglion cells; nrf-2, ARE, HO-1 and γ-GCS expression in neuroblastoma cells treated with pinocembrin; MDA and PC in rats given propolis; nNOS, iNOS and glutathione in ischemic rats given pinocembrin; oxidative stress in liver and brain, DNA fragmentation in hepatic encephalopathy induced rats given propolis; brain SOD, CAT, GSH and MDA in brain damaged rats; myeloperoxidase, TNF-α, NF-kB, IL-6 and IL-10 in ischemic rats
[20,24,39]	8	Gut Health. Outcomes: Human faecal in vitro fermentations of rutin; gut bacterial cell viability and production of SCFAs after propolis administration; parasite infected mice administered propolis
[14,32,34,55]	36	Immune System Effects. Outcomes: mononuclear cell response to exercise following caffeic-acid phenethyl-ester ingestion; liver, kidney and immune cell response to propolis; splenic NK cytotoxic, T lymphocyte proliferation and antibody generation cells, as well as the phagocytosis of peritoneal macrophages, ear swelling and serum contents of IgG; IgM in mice within propolis vs. control
[9,10,11,12,17,18,21,26,27,28,33,35,37,38,45,46,47,48,49,50,51,52,53,54,55,56,57,58,59,60,61,62,63]	204	Neuroprotection. Outcomes: Antioxidant enzymes, lipid peroxidation and infarct volume were measured post stroke in mice; toxicity in PC12 cells; behavioural performance of Dgal-treated mice; Cell viability and apoptosis in neuronal cells pretreated with pinocembrin and glutamate; Blood-brain barrier disruption was measured in rats with cerebral ischemia; cognitive test performance in rats given propolis; cell viability loss, apoptotic rate and decreased Bcl-2/Bax ratio in neuroblastoma cells; expression level of neurotrophic factors in dental pulp cells; oxidative stress in human neuroblastoma cells; induced seizures in rats given propolis; neurological ability and cognition in ischemic rats given pinocembrin; iNOS expression in hepatic encephalopathy induced rats given propolis; brain damaged rats behavioural assessment; cerebral edema in rats
[9,10,11,12,14,15,16,19,20,22,24,26,27,28,29,30,31,32,33,34,35,37,38,39,41,45,46,47,48,49,52,57,59,60,61,62,63]	44	Study Design. Outcomes: Propolis compound identification; animal vs. human intervention; intervention length in human trials typically 4 weeks.; animal models of disease; gene expression profile following propolis administration; phenolic compounds; blood brain barrier uptake of pinocembrin
[24,31]	8	Wound Healing and Skin Protection. Outcomes: Propolis and its protective effects on hydrogen peroxide-induced changes in mouse fibroblast cells; antimicrobial, antioxidant and anti-inflammatory properties in humans

Key: IL-6: interleukin-6; TNF-α: tumor necrosis factor-alpha; NF-kB: nuclear factor kappa-B; HDL: high-density lipoprotein; ALT: alanine aminotransferase; ALP: alanine phosphatase; CK: creatine kinase; MDA: malondialdehyde; nrf-2: nuclear factor erythroid 2-related factor 2; ARE: antioxidant response element; HO-1: hemeoxygenase-1; γ-GCS: gamma-glutamylcysteine synthetase; PC: protein carbonyl; nNOS: neuronal nitrite oxide synthase; iNOS: inducible nitric oxide synthase; CAT: catalase; GSH: glutathione; IL-10: interleukin-10; SCFAs: short-chain fatty-acids; NK: natural killer; IgG: immunoglobulin G; IgM: immunoglobulin M; Bax: apotosis regulator.

## Supplementary Materials

The following are available online at http://doi.org/10.5281/zenodo.3334965, Figure S1: PRISMA Search Chart, Table S1: Supplementary Data S1 Table S1 Search strategy.
